# Knockdown of UbcH10 Enhances the Chemosensitivity of Dual Drug Resistant Breast Cancer Cells to Epirubicin and Docetaxel

**DOI:** 10.3390/ijms16034698

**Published:** 2015-03-02

**Authors:** Cheng Wang, Yun-Hao Pan, Ming Shan, Ming Xu, Jia-Lin Bao, Li-Ming Zhao

**Affiliations:** 1 Department of Breast Surgery, Central Hospital of Huangpu District, Shanghai 20002, China; E-Mails: panyunhao@gmail.com (Y.-H.P.); shanming99@gmail.com (M.S.); huangpuruxian@163.com (J.-L.B.); 2 Department of Pathology, Central Hospital of Huangpu District, Shanghai 20002, China; E-Mail: xujshan@sina.com; 3 Department of Respiratory Disease, Eastern Hepatobiliary Surgery Hospital, Secondary Military Medical University, Shanghai 200433, China

**Keywords:** UbcH10, chemosensitivity, breast cancer, gene knockdown

## Abstract

Breast cancer is one of the most common and lethal cancers in women. As a hub gene involved in a diversity of tumors, the ubiquitin-conjugating enzyme H10 (UbcH10), may also play some roles in the genesis and development of breast cancer. In the current study, we found that the expression of UbcH10 was up-regulated in some breast cancer tissues and five cell lines. We established a dual drug resistant cell line MCF-7/EPB (epirubicin)/TXT (docetaxel) and a lentiviral system expressing UbcH10 shRNA to investigate the effects of UbcH10 knockdown on the chemosensitivity of MCF-7/EPB/TXT cells to epirubicin and docetaxel. The knockdown of UbcH10 inhibited the proliferation of both MCF-7 and MCF-7/EPB/TXT cells, due to the G1 phase arrest in cell cycle. Furthermore, UbcH10 knockdown increased the sensitivity of MCF-7/EPB/TXT cells to epirubicin and docetaxel and promoted the apoptosis induced by these two drugs. Protein detection showed that, in addition to inhibiting the expression of Ki67 and cyclin D1, UbcH10 RNAi also impaired the increased BCL-2 and MDR-1 expression levels in MCF-7/EPB/TXT cells, which may contribute to abating the drug resistance in the breast cancer cells. Our research in the current study demonstrated that up-regulation of UbcH10 was involved in breast cancer and its knockdown can inhibit the growth of cancer cells and increase the chemosensitivity of the dual drug resistant breast cancer cells to epirubicin and docetaxel, suggesting that UbcH10 may be a promising target for the therapy of breast cancer.

## 1. Introduction

Currently, breast cancer is one of the most common cancers in women, and surgery combined with chemotherapy has been frequently used in the treatment of breast cancer. Though the routine therapy may increase the patient survival rate, improvement in patient quality of life is limited. With research advances in molecular biology and gene functions in tumors, we know more about the molecular mechanisms of pathogenesis of breast cancer. The genesis and development of breast cancer is a multiple-step process caused by the activation or deactivation of a number of cancer-associated genes at different points in time and space through various pathways. Increasing numbers of genes involved in breast cancer have been found, including *Ets-1* (E-twenty-six-1), *PKC* (protein kinase C), *CEP4* (CDC42 effector protein 4), *Her2* (erb-b2 receptor tyrosine kinase 2), *ALDH1* (aldehyde dehydrogenase 2), *FOXA1* (forkhead box A1) and *HOXD10* (homeobox D10) [1,2,3,4,5,6].

Due to its own characteristics, the surgical removal of breast cancer inevitably influences the life quality of patients to some extent, so the use of chemotherapy in the treatment of early breast cancer is of critical importance. Given that more breast cancer-related genes are being identified, gene therapy has gradually become a research focus in the treatment of breast cancer. Through gene intervention, gene target therapy not just directly regulates the genesis and development of breast cancer, but increases the effects of chemotherapy. The different sensitivity of patients sustaining breast cancer due to diverse pathogenic mechanisms, individual differences, and the resistance of chemotherapy severely restricts the clinical outcome of chemotherapy. A variety of genes have been found to play important roles in the resistance and sensitivity of breast cancer to drugs, such as *KIC19*, *TYPH*, *HSP17*, *c-met*, *ERK1/2*, and *Caveolin-1* [7,8,9,10]. Research on resistance mechanisms have indicated that the abnormal expression levels of tumor-associated genes may be the root cause leading to the diverse sensitivity of different patients to the same chemotherapeutic drug, and decreased effects on the same patient with continued drug administration. Therefore, searching for new critical genes regulating breast cancer, and identifying the roles of specific genes in the genesis and development of breast cancer has become an important direction in the field of breast cancer treatment.

As an important pathway for protein modification and degradation, the ubiquitin-mediated protein degradation, utilizing ubiquitin-activating enzyme, ubiquitin-conjugating enzyme, ubiquitin ligase and the proteasome, plays an important role in the cell cycle. Recently, it was reported that the ubiquitin-conjugating enzyme H10 (UbcH10), also known as Ubiquitin-conjugating Enzyme E2C (UBE2C), is closely related to the genesis and development of multiple cancers [11,12]. Despite several studies identifying UbcH10 as E2 of the anaphase-promoting complex (APC) for the degradation of cyclin A and B in cell cycle control, the definite mechanism how UbcH10 is related to tumorigenesis remains unclear. Perotta *et al.* found that UbcH10 was highly expressed in lung cancers, and may be used as a marker for malignancy grading in tumors [13]. UbcH10 can also be used as a prognostic indicator in surgical treatment of bladder cancer [14]. In addition, silencing UbcH10 with RNAi can inhibit intestinal cancer *in vitro* and *in vivo* [15]. However, there are few reports on the research of the relation between UbcH10 and drug resistance in breast cancer. Zhao *et al.* have reported that abnormal expression of UbcH10 in lung cancers enables it to be used as a candidate grading marker and inhibition of UbcH10 can increase the sensitivity of lung cancer cells to chemotherapeutics [16]. Few studies on UbcH10 and breast cancer have been reported. Berlingieri *et al.* demonstrated that UbcH10 expression was positively correlated with Ki-67, and the inhibition of ErbB2 in breast cancer cells can reduce UbcH10 level. The possibility of treatment of breast cancer with *UbcH10* gene interference has also been discussed [17]. Fujita *et al.* have demonstrated the correlation between UbcH10 and breast cancer through clinical pathology, human cancer array and biochemical analysis [18]. Their results showed that positive rate of UbcH10 expression was higher in breast cancer tissues in comparison with adjacent non-malignant tissues, and UbcH10 expression correlates with the tumors of increased histological grade. Therefore, abnormal UbcH10 may disturb the normal cell cycle progress, resulting in aggression of cancer cells. However, there is no report on the relation between UbcH10 and drug resistance and chemotherapeutic sensitivity in breast cancer.

In the current study, we examined UbcH10 expression in clinical breast cancer samples, and verified the data of breast cancer samples in several breast cancer cell lines, by comparing expression of UbcH10 in normal and breast cancer cell lines. On this basis, we established a breast cancer cell line resistant to both epirubicin (EPB) and docetaxel (TXT) through gradual induction, and evaluated the difference in UbcH10 expression between the resistant cell line and its parent cell line. We used our lentiviral system to silence *UbcH10* gene in the resistant cell lines and the parent cell line, and examined their proliferation, cell cycle distribution, sensitivities to chemotherapeutics, and apoptosis, to evaluate the effects of gene inference on tumor cells, and to investigate the possibility of using the *UbcH10* gene as a therapeutic target in breast cancer.

## 2. Results and Discussion

### 2.1. Analysis of Differential Expression of Ubiquitin-Conjugating Enzyme H10 (UbcH10) in Breast Cancer Tissues and Cell Lines

**Figure 1:**
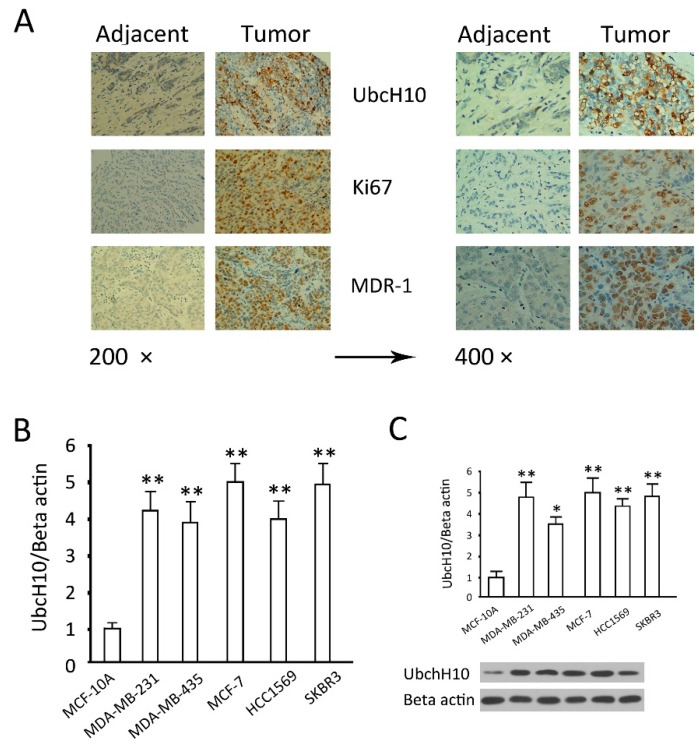
Expression of ubiquitin-conjugating enzyme H10 (UbcH10) in breast cancer tissues and cell lines. (**A**) Representative photos of immunohistochemical staining of UbcH10, Ki67 and P-glycoprotein (MDR-1) in adjacent tissues and breast tumor tissues; (**B**) mRNA levels of UbcH10 expressed in the indicated cell lines evaluated by quantitative PCR; and (**C**) Protein levels of UbcH10 were evaluated by Western blotting in the indicated cell lines: **Upper**: The optical density of the target band divided by the optical density of the β-actin band; **Lower**: Representative blots. Data are expressed as mean ± standard deviation (SD) of at least three independent experiments. * *p* < 0.05 and ** *p* < 0.01, when compared to the MCF-10A group.

Based on our previous research [19], we examined UbcH10, Ki 67 and MDR1 in low-differentiated breast cancers and adjacent tissues. Immunohistochemical results showed that, in comparison with adjacent tissues, the expression levels of UbcH10, as well as Ki67 and MDR1, were increased in cancer tissues (Figure 1A). The UbcH10 mRNA and protein content analysis in five breast cancer cell lines showed that UbcH10 mRNA expression in the breast cancer cells was significantly higher than that in the normal mammary epithelial cells (*p* < 0.05) (Figure 1B). The results of western blotting of UbcH10 coincided exactly with the results of mRNA. These results suggest that UbcH10, as well as the tumor markers, Ki67 and MDR1 are closely associated with breast cancer. According to these data, it would be interesting to understand whether UbcH10 plays an important role in the viability and sensitivity to chemotherapeutics of breast cancer cells, and whether UbcH10 knockdown at the transcriptional level has a profound impact on the genesis and development of breast cancer and chemotherapeutics. In particular, the MCF-7 cell line, one of the cell lines with the highest UbcH10 levels, was selected for the following experiments *in vitro*.

### 2.2. Establishment and Verification of the Dual Drug Resistant Cell Line (MCF-7/EPB/TXT)

**Figure 2:**
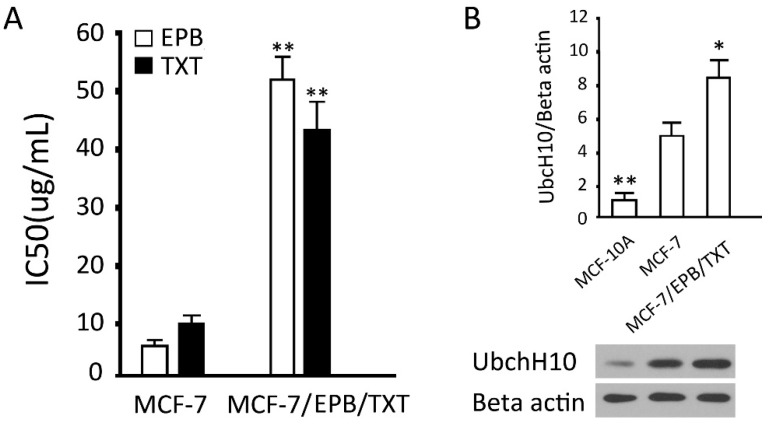
Establishment of the dual drug resistant cell (MCF-7/EPB/TXT) line and its expression of UbcH10 protein. (**A**) IC_50_ of EPB and TXT for MCF-7 and MCF-7/EPB/TXT cells; and (**B**) Protein levels of UbcH10 were assessed by western blotting in the indicated cell lines: **Upper**: The optical density of the target band divided by the optical density of the β-actin band; **Lower**: Representative blots. Data are expressed as mean ± SD of at least three independent experiments.* *p* < 0.05 and ** *p* < 0.01, when compared to the corresponding MCF-7 group.

We successfully established a dual drug resistant cell line MCF-7/EPB/TXT using gradual induction. For 24 h exposure, the the half maximal (50%) inhibitory concentration (IC_50_) values of epirubicin (EPB) and docetaxel (TXT) for MCF-7 cells were 5.5 ± 0.11 and 10.8 ± 0.19 μg/mL, and those for MCF-7/EPB/TXT cells were 52.1 ± 5.02 and 42.6 ± 6.84 μg/mL, increased 9.5- and 3.9-fold, respectively. There were significant differences between the two groups (*p* < 0.01) (Figure 2A). The results of the UbcH10 protein detection showed a significant increase of UbcH10 in MCF-7/EPB/TXT (*p* < 0.05), in comparison with the parent MCF-7 cells (Figure 2B). Combined with the MDR-1 protein in breast cancer tissues, the results indicated that UbcH10 was closely related to the sensitivity of breast cancer cells to chemotherapeutics.

### 2.3. Detection of Gene Transfection Efficiency and Screening of Effective siRNA Sequence

**Figure 3:**
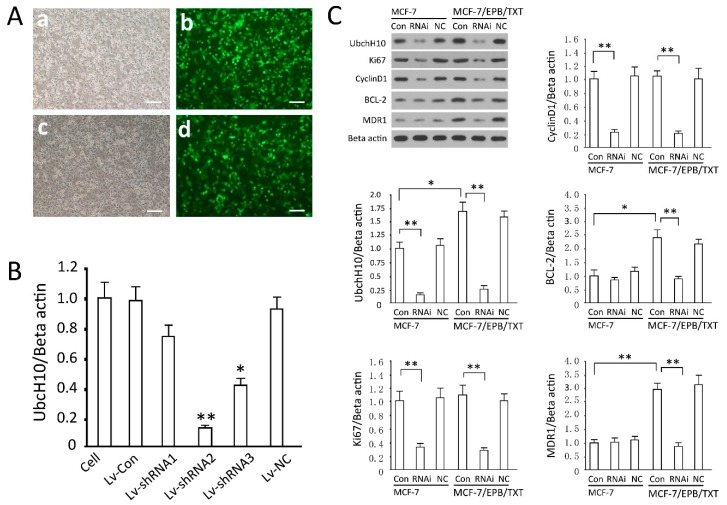
Effective knockdown of UbcH10 expression and effects of UbcH10 knockdown on other proteins. (**A**) MCF-7 and MCF-7/EPB/TXT cells were infected with Lv-shRNA2 and GFP was observed under fluorescence microscopy 72 h later, scale bars =50 μm. (**a**,**b**) MCF-7 cells were infected with Lv-Con; (**c**,**d**) MCF-7 cells were infected with Lv-shRNA2; (**B**) MCF-7 cells were infected with Lv-Con, Lv-NC, or Lv-shRNA1-3 and mRNA levels of UbcH10 were assessed by quantitative PCR. Data are expressed as mean ± SD of at least three independent experiments. * *p* < 0.05 and ** *p* < 0.01, when compared to the cell group; and (**C**) Effects of UbcH10 knockdown on Ki67, cyclin D1, Bcl-2, and MDR1: Representative blots and the optical density of the target band divided by the optical density of the β-actin band. Data are expressed as mean ± SD of at least three independent experiments. * *p* < 0.05 and ** *p* < 0.01.

High efficiency of gene transfer can be attained through the lentiviral system in MCF-7 and MCF-7/EPB/TXT cells. According to the data of green fluorescent protein (GFP) expression, the gene delivery efficiency was close to 100% (Figure 3A). UbcH10 mRNA detection in the cells demonstrated that all three siRNA sequences can inhibit UbcH10 expression, and siRNA2 was most potent, reducing UbcH10 mRNA by 82.7% (*p* < 0.01, *vs.* the uninfected group). On the contrary, the viral control group and negative control (NC) sequence did not show statistically significant effects on the target genes (*p* > 0.05) (Figure 3B). It is difficult to transfect the breast cancer cells using conventional approaches, but efficient gene delivery is a premise for gene knockdown that is crucial to genetic studies. The lentiviral system was employed in the study, which solved the problem well, since it can knock down the *UbcH10* gene in MCF-7 cells in an efficient, permanent and stable way. In the following experiments, the recombinant virus expressing siRNA2 was used to silence *UbcH10*.

### 2.4. Effects of UbcH10 Knockdown on Relevant Protein Contents

**Figure 4:**
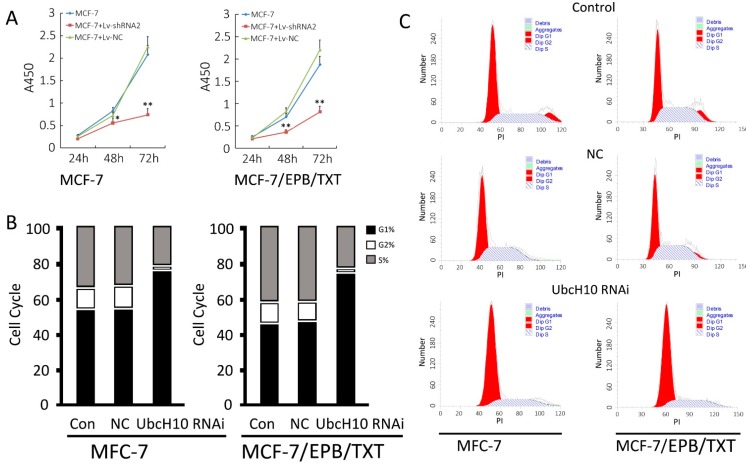
UbcH10 knockdown inhibits proliferation and changes cell cycle distributions in MCF-7 and MCF-7/EPB/TXT cells. (**A**) Growth curves of MCF-7 and MCF-7/EPB/TXT cells before and after *UbcH10* gene knockdown. Data are expressed as mean ± SD of at least three independent experiments. * *p* < 0.05 and ** *p* < 0.01, when compared to the control cell group; (**B**) Cell cycle distribution in percentages of different groups. Data were obtained by flow cytometry and presented as mean (*n* = 3). Black bars represent the percentages of G1 fractions, white bars represent the percentage of G2 fraction, and the grey bars represent the percentage of S fraction; and (**C**) Representative plots of MCF-7 and MCF-7/EPB/TXT cells or those infected with Lv-NC or Lv-UbcH10 RNAi.

By comparing the effects of UbcH10 silencing on proliferation, apoptosis, and drug-resistance related proteins in MCF-7/EPB/TXT and MCF-7, we found some interesting phenomena: There was no significant difference in Ki67 and cyclin D1 between MCF-7/EPB/TXT and MCF-7 cells (*p* > 0.05, Figure 3C); Ubch10 knockdown inhibited the expression of Ki67 and cyclin D1 in both cell groups, consistent with the results of proliferation and cell cycle detection (Figure 4); the expression levels of BCL-2 and MDR-1 in MCF-7/EPB/TXT cells were significantly higher than those in MCF-7, coincident with the production of drug resistance. In MCF-7 cells, UbcH10 silencing did not inhibit the expression of BCL-2 and MDR-1, but significantly inhibited their expression in MCF-7/EPB/TXT cells (*p* <0.05, Figure 3C); in terms of all of the detected indexes, there was no difference between the NC group and the control group (*p* > 0.05, Figure 3C).

### 2.5. Effects of UbcH10 Knockdown on Proliferation and Cell Cycle in MCF-7 and MCF-7/EPB/TXT Cells

By comparing the growth curves of MCF-7 and MCF-7/EPB/TXT cells before and after *UbcH10* gene knockdown, UbcH10 silencing seemed to significantly inhibit logarithmic phase proliferation in both the drug-resistant and parent cell lines. The cell numbers at 48 or 72 h in gene knockdown groups were significantly lower than the control groups (*p* < 0.05), and there was no observable difference between the NC group and the cell group (*p* > 0.05) (Figure 4A). The results of cell cycle detection indicated that UbcH10 blocked cells at G1 phase, both in the drug-resistant and parent cell lines. These results suggest that UbcH10 has a direct impact on the proliferative activity of breast cancer and the inhibition of proliferation was not affected by the drug resistant mechanism.

### 2.6. Effects of UbcH10 Knockdown on Sensitivity of Resistant Cells to Chemotherapeutics

**Figure 5:**
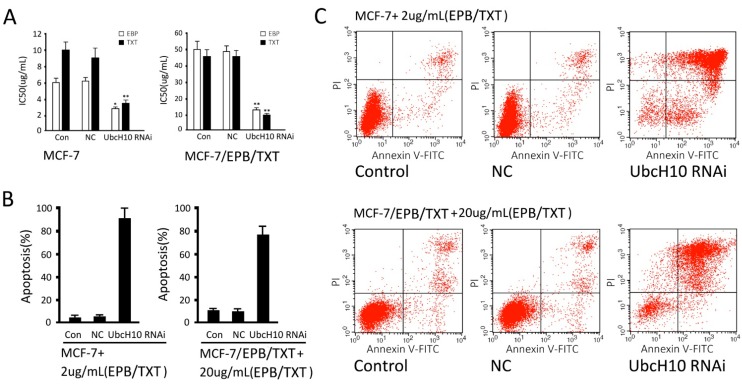
UbcH10 knockdown increases the sensitivity of resistant cells to chemotherapeutics. (**A**) IC_50s_ of EPB and TXT for MCF-7 and MCF-7/EPB/TXT cells. Data are expressed as mean ± SD of at least three independent experiments. * *p* < 0.05 and ** *p* < 0.01, when compared to the Con group; (**B**) Apoptosis caused by EPB and TXT in MCF-7 and MCF-7/EPB/TXT cells; and (**C**) Representative plots of MCF-7 and MCF-7/EPB/TXT cells with or without UbcH10 knockdown treated with EBP/TXT.

Silencing UbcH10 in the resistant cells and its parent cells evidently increased their sensitivity to chemotherapeutics. For MCF-7 cells, IC_50_ values of EPB and TXT were decreased from 5.81 ± 0.24 and 9.78 ± 0.62 to 2.68 ± 0.16 and 3.12 ± 0.42 μg/mL (*p* < 0.05, *vs.* the control group); but there was no significant difference between the NC group and control group. For MCF-7/EPB/TXT cells, IC_50_ values of EPB and TXT were decreased from 48.85 ± 5.06 and 45.15 ± 4.04 to 12.12 ± 0.18 and 8.96 ± 0.21 μg/mL (*p* < 0.05, control group), and no significant difference was detected between the NC group and control group (Figure 5A). Furthermore, the results from apoptosis detection indicated UbcH10 silencing increased drug-induced apoptosis. For MCF-7 cells, the treatment with 2 μg/mL EPB and TXT for 24 h resulted in an apoptosis rate of 6.88% ± 0.87% in the cell control group, but a rate of 90.23% ± 10.12% in the UbcH10 knockdown group; there was a significant difference between the UbcH10 knockdown group and the control group (*p* < 0.05) but not between the NC group and the control group (*p* > 0.05); The same trend was found in MCF-7/EPB/TXT cells: The treatment with 20 μg/mL EPB and TXT for 24 h resulted in an apoptosis rate of 10.12% ± 1.23% in the resistant cell control group, but a rate of 80.52% ± 8.69% in the UbcH10 knockdown group; there was a significant difference between the UbcH10 knockdown group and the control group (*p* < 0.05) but not between the NC group and the control group (*p* > 0.05) (Figure 5B,C). The results mentioned above suggested that the UbcH10 knockdown could increase the sensitivity of breast cancer cells to EPB and TXT in the resistant cells and the parent cells.

The ubiquitin-proteasome proteolytic pathway is one of the important paths for degradation of proteins in eukaryotic cells, and closely related to proliferation, apoptosis, and other biological phenomena. One of the E2 protease family members, UbcH10 plays important roles in ubiquitylation, and is closely related to the genesis and development of tumors, according to a number of studies. It is overexpressed in tumor tissues derived from a variety of tissues [20,21]. As a hub gene expressed abnormally in a number of tumors, UbcH10 may also be involved in the genesis and development of breast cancer. Therefore, the investigation in the molecular physiopathologic mechanism extends our knowledge of how UbcH10 is associated with breast cancer and may help to understand the malignant biological behavior of breast cancer and provide new targets for the clinical treatment of breast cancer.

In the present study, we found that UbcH10 levels in tumor tissues were higher than those in para-carcinoma tissues and were related to the expression of Ki67, according to immunohistochemical results. The analysis in five breast cancer cell lines and the normal mammary epithelial cell line showed that UbcH10 was also higher in breast cancer cells. The lentiviral system was employed to knock UbcH10 down in MCF-7 cells, and found that UbcH10 knockdown inhibited the proliferation of MCF-7 cells, and resulted in G1 arrest. Taking into consideration that UbcH10 knockdown increased the sensitivity of lung cancer cells to chemotherapeutics, we established a dual resistant cell line, MFC-7/EPB/TXT, and knocked UbcH10 down using the lentiviral system. The results confirmed the hypothesis. UbcH10 affected the proliferation of tumor cells and their corresponding resistant cell line. Moreover, in the resistant cells, UbcH10 knockdown also increased the sensitivity to chemotherapeutic drugs. These results indicated that UbcH10 intervention may be used for gene therapy whether chemotherapy has been implemented or not. In addition, potential drugs that inhibit UbcH10 directly may also be used in combination therapy of resistant breast cancers, though further studies are required.

EPB and TXT are commonly used in treatment of early breast cancer [22,23,24]. To attain a better outcome, multiple drugs are frequently used in combination. But the sensitivity of breast cancer to different drugs varies, and drug resistance greatly reduces the effects of chemotherapy [25,26]. Since both EPB and TXT are substrates for P-glycoprotein (P-gp/MDR1) [27,28], our results showed UbcH10 silencing decreased MDR1 expression level in MCF-7/EPB/TXT cells, which may enhance the sensitivity of these cells to chemotherapy. In addition, UbcH10 silencing also changed BCL-2 levels in MCF-7/EPB/TXT cells, possibly impairing the resistance of these cells to drugs. If we can find a gene which is directly related to the viability of tumor cells and can affect their sensitivity to chemotherapeutic drugs, a milestone will made in the treatment of breast cancer. Fortunately, our research unveiled that UbcH10 had a great potential-silencing of the *UbcH10* gene that not only inhibited the proliferation of breast cancer cells, but increased sensitivity to EPB and TXT. More importantly, the effects of UbcH10 remained in the resistant cells. These results suggest to us that UbcH10 interference has a profound effect on the treatment of breast cancer, and that chemotherapy and the development of drug resistance does not affect the role of UbcH10.

## 3. Experimental Section

### 3.1. Analysis on Differential UbcH10 Expression in Tumor and Adjacent Tissues from Breast Cancer Patients

Twenty samples of breast cancer and corresponding adjacent tissues were collected (Central Hospital of Huangpu District, Shanghai, China), fixed with 4% paraformaldehyde, embedded into paraffin, sliced and subject to conventional immunohistochemical detection of UbcH10, Ki67 and MDR1. All primary and secondary antibodies used were purchased from Abcam (Cambridge, MA, USA). The pathological results were determined by a researcher and reviewed by two pathologists (Ming Xu, and Weiqing Xu, Central Hospital of Huangpu District, Shanghai, China).

### 3.2. Examination of UbcH10 Expression in Breast Cancer Cell Lines

Breast cancer cell lines, including MDA-MB-231, MDA-MB-435, MCF-7, HCC1569, and SKBR3, and a normal mammary epithelial cell line MCF-10A (purchased from Cell Bank, Chinese Academy of Science) were cultured in RPMI1640, supplemented with 10% fetal bovine serum (FBS), at 5% CO_2_ and 37 °C. Total RNA and protein were extracted from the cells in logarithmic phase. Real-time PCR and western blotting were used to detect mRNA and protein levels of UbcH10.

### 3.3. Establishment of EPB and TXT Resistant Cell Line

EPB and TXT were purchased from Sigma (St. Louis, MO, USA), and dissolved in absolute ethanol to prepare stock solutions (1 mg/mL). MCF-7 cells were cultured under normal conditions, and were reseeded at 1:5 when the confluence reached 80%. Cells were maintained in the complete medium containing 10% FBS. EPB and TXT were added into the medium, and the final concentration was increased gradually (1 to 2, 2 to 5, 5 to 10, 10 to 20, 20 to 30, 30 to 50, 50 to 70, and 70 to 100 μg/mL, increased each every two passages). The cells were cultured for three more passages after the concentration reached 100 μg/mL. The cells in logarithmic phase were collected and reseeded into 96-well plates. Drugs were added to final concentrations of 1, 5, 10, 20, 50 and 100 μg/mL. After an incubation of 24 h, cell viabilities were accessed using CCK-8 (DOJINDO, Kumamoto, Japan), for calculation of inhibitory ratio and IC_50_, to identify whether the induction experiments were successful. Meanwhile, the resistant cells and the parent cells were collected, and subject to total protein extraction and western blotting detection of UbcH10.

### 3.4. Screening of Effective siRNA Sequence and Intervention of UbcH10

In accordance with the gene data of UbcH10 (NM_007019.2), on-line siRNA design software was used to design three shRNA sequences: siRNA1, 5'-GACCATCCATGGAGCAGCT-3'; siRNA2, 5'-GTTCCTCACGCCCTGCTAT-3'; and siRNA3, 5'-GGAAAAGTGGTCTGCCCTG-3'; and 5'-CTCCCGTCATGTGCTTCAC-3' was used as the negative control (NC) sequence. The complementary long chain DNA sequences containing BamHI and EcoRI cleavage sites with a loop of 5'-CTTCCTGTCAGA-3' were synthesized, annealed and inserted into the linear vector (System Bioscience, Mountain View, CA, USA) to construct the recombinant shRNA expression vector. The correct shRNA expression vectors confirmed by sequencing analysis were amplified and purified using an endotoxin-free plasmid extract kit (Qiagen, Hilden, Germany). The expression vectors and viral package plasmid mixture (System Bioscience) were transfected into 293T cells (System Bioscience). The viral solution was collected 48 h later and the viral titer was determined by gradient dilution. MCF-7 and MCF-7/EPB/TXT cells in logarithmic phase were infected with the virus at an multiplicity of infection (MOI) of 10. The infection efficiency was assessed based on the expression of green fluorescent protein (GFP). The cells were collected 72 h after viral infection, and the total protein was extracted for detection of UbcH10.

### 3.5. Effects of UbcH10 Gene Silencing on Proliferation and Cell Cycle Distribution of MCF-7 and MCF-7/EPB/TXT Cells

The MCF-7 and MCF-7/EPB/TXT cells infected by the recombinant lentiviruses were made into cell suspension by trypsinization and seeded into 96-well plates at 5 × 10^3^ cells per well solution. The cells were cultured under normal conditions, and the viabilities were examined using CCK-8. The cell suspensions were also seeded into 24-well plates, at 1 × 10^5^ cells per well and cultured under normal conditions. The cells were collected and subjected to cell cycle analysis 24 h later: The cells were washed with Dulbecco’s Phosphate-Buffered Sallines (dPBS) twice, re-suspended with 500 μL binding buffer, 5 μL propidium iodide added, incubated at room temperature in the dark for 5 min and detected by flow cytometry.

### 3.6. Detection of Effects of UbcH10 Gene Interference on Sensitivity of Resistant Cells to Chemotherapeutics

Seventy-two hours after infection, the MCF-7 and MCF-7/EPB/TXT cells were made into cell suspensions by trypsinization and seeded into 96-well plates at 5 × 10^4^ cells per well. EPB and TXT were added to final concentrations of 1, 10, 50 and 100 μg/mL. The cells were incubated under normal conditions for 24 h, and the viabilities were examined using CCK-8. The absorbance values were used to calculate the inhibition ratios and then the changes in IC_50_ at 24 h.

Meanwhile, apoptosis was measured to evaluate the changes in sensitivity of the cells to these two drugs. The cell suspensions were inoculated into six-well plates, 2 mL per well. After the cells were incubated for 12 h under normal conditions, the drugs were added to a final concentration of 20 μg/mL for the drug resistant group and 2 μg/mL for the parent group. The cells were maintained for 48 h and collected for apoptotic assay with Annexin V:FITC apoptosis detection kit II (BD Biosciences, San Jose, CA, USA). The cells were washed with dPBS twice, and resuspended in 500 μL binding buffer with 5 μL Annexin V-FITC in the dark for 10 min. The cells were then stained with 5 μL propidium iodide (PI) for 5 min. Apoptosis was analyzed at an excitation wavelength at 488 nm using the FL1 channel for Annexin V-FITC and the FL2 channel for PI.

### 3.7. Effects of UbcH10 Gene Silencing on Relevant Protein Contents

After analyzing the effects of *UbcH10* gene silencing on the proliferation and chemotherapeutic sensitivity of MCF-7 and MCF-7/EPB/TXT cells, we made a preliminary analysis on the cause of the changes. And we selected a number of genes for further investigation, including *Ki67* and *cyclin D1* associated with cell proliferation and cycle, BCL-2, an apoptosis related gene, and *MDR-1*, a gene involved in drug resistance. Both MCF-7 and MCF-7/EPB/TXT cells were divided into the control group, NC group and UbcH10 knockdown group. The changes in protein contents caused by UbcH10 gene silencing were measured by western blotting, for the preliminary analysis on the regulatory mechanism of UbcH10 on breast cancer cells.

### 3.8. Detection of UbcH10 mRNA by Real-Time PCR

Total RNA was extracted from the cells and reverse transcribed into cDNA with oligo dT18. The RNA contents were detected by the fluorescence dye method strictly following the manufacturer’s instructions of the kit. Relative mRNA content was analyzed using the 2^ΔΔ*C*t^ method, with β-actin (NM_001101.3) as the referral gene. The primers used for PCR amplification were: β-actin-forward primer: 5'-CCTGTACGCCAACACAGTGC-3' and β-actin-reverse primer: 5'-ATACTCCTGCTTGCTGATCC-3', producing a segment of 211 bp; UbcH10-forward primer: 5'-ACCCTCATGATGTCTGGCGATAAA-3' and UbcH10-reverse primer: 5'-GTGATAGCAGGGCGTGAGGAACTT-3', producing a segment of 192 bp. The PCR reaction system was: Takara SYBR Premix Ex Tap 10 μL, forward and reverse primers (20 μM) 0.2 μL each, cDNA 2 μL, and dH_2_O added to 20 μL. The reaction parameters were: 40 cycles of denature at 95 °C for 5 s, anneal at 60 °C for 20 s, and elongation at 72 °C for 20 s.

### 3.9. Detection of Relative Protein Content by Western Blotting

About 1 × 10^7^ cells were collected and lysed in 1 mL cell lysis buffer (50 mM pH 8.0 Tris, 1 mg/mL leupeptin, 150 mM NaCl, 0.5% Nonidet P-40, 5 mM EDTA, 100 mM phenylmethylsulfonyl fluoride, 1 M dithiolthretol, and 1 mg/mL aprotinin) for protein extraction. The protein concentrations were determined by BCA assay (Pierce, Rockford, IL, USA). Protein samples (10 μL/lane) were separated by 10% SDS-PAGE and transferred to polyvinylidene fluoride (PVDF) membranes using wet transfer (400 mA, 90 min). The members were blocked in tris-buffered saline and tween 20 (TBST) containing 5% nonfat milk at room temperature for 2 h and incubated with the primary antibodies (UbcH10, 1:500; Ki67, 1:400; cyclin D1, 1:800; BCL-2, 1:600; MDR-1, 1:200; β actin, 1:500, all purchased from Santa Cruz Biotechnology, Santa Cruz, CA, USA) at 4 °C overnight. The blots were then rinsed with TBST three times and incubated with the secondary antibody (goat anti-mouse IgG, 1:3000, Santa Cruz Biotechnology) for 2 h. Electro-chemi-luminescence (ECL) chemiluminescence substrates and X-ray film were used to detect the bands, and relative optical densities were analyzed with image processing software (NatureGene Corp., Beijing, China): Relative contents of UbcH10 were equal to the optical density of the target band divided by the optical density of the β-actin band.

### 3.10. Detection of Cell Proliferation Using CCK-8

The cells were inoculated into 96-well plates and treated according to the experimental grouping requirements. CCK-8 (10 μL/well) solution was added, and the cells were maintained at 37 °C and 5% CO_2_ for 4 h, and the absorbance (optic density, OD) at 450 nm was measured, which was calibrated with the results from the wells that contain known numbers of viable cells prepared and processed as the same.

### 3.11. Statistical Analysis

SPSS13.0 (SPSS, Chicago, IL, USA) was used for statistical analysis. All data are expressed as mean ± standard deviation (SD). Factorial analysis was employed for inter-group and intra-group comparison; *p* values < 0.05 were taken as the level of significance.

## 4. Conclusions

The protein content analysis showed that UbcH10 knockdown suppressed the expression of Ki67 and cyclin D1, and thus influenced the proliferation and cell cycle in cancer cells. Meanwhile, silencing UbcH10 significantly impaired the increase in BCL-2 and MDR-1 induced by chemotherapeutic drugs, which may partly account for the increased sensitivity of the dual drug resistant cell line. Certainly, as a protein involved in ubiquitylation, UbcH10 may affect many tumor-associated proteins, and affect tumors in different ways. But the direct inhibition on proliferation and increase in sensitivity by UbcH10 may have guiding significance in the research in and the treatment of breast cancer. The unchanged effects of UbcH10 in drug resistant breast cancer cells and parent cells suggest that targeted gene interference may exert effects in the whole process of chemotherapy. Further study is required to investigate the molecular mechanisms by which UbcH10 knockdown reduces cell proliferation and increases chemosensitivity.
